# Neonatal monocytes demonstrate impaired homeostatic extravasation into a microphysiological human vascular model

**DOI:** 10.1038/s41598-020-74639-z

**Published:** 2020-10-20

**Authors:** Guzman Sanchez-Schmitz, Elena Morrocchi, Mitchell Cooney, Dheeraj Soni, Rahima Khatun, Paolo Palma, David J. Dowling, Ofer Levy

**Affiliations:** 1 Division of Infectious Diseases, Boston Children’s Hospital, Boston, MA USA; 2 Precision Vaccines Program, Boston Children’s Hospital, Boston, MA USA; 3 Harvard Medical School, Harvard University, Boston, MA USA; 4 Broad Institute of Harvard and MIT, Cambridge, MA USA; 5 Academic Department of Paediatrics (DPUO), Research Unit of Congenital and Perinatal Infections, Children’s Hospital Bambino Gesù, Rome, Italy; 6 Chair of Paediatrics, Department of Systems Medicine, University of Rome Tor Vergata, Rome, Italy

**Keywords:** Ageing, Immunology, Innate immune cells

## Abstract

Infections are most frequent at the extremes of life, especially among newborns, reflecting age-specific differences in immunity. Monocytes maintain tissue-homeostasis and defence-readiness by escaping circulation in the absence of inflammation to become tissue-resident antigen presenting cells in vivo. Despite equivalent circulating levels, neonates demonstrate lower presence of monocytes inside peripheral tissues as compared to adults. To study the ability of monocytes to undergo autonomous transendothelial extravasation under biologically accurate circumstances we engineered a three-dimensional human vascular-interstitial model including collagen, fibronectin, primary endothelial cells and autologous untreated plasma. This microphysiological tissue construct enabled age-specific autonomous extravasation of monocytes through a confluent human endothelium in the absence of exogenous chemokines and activation. Both CD16− and CD16+ newborn monocytes demonstrated lower adherence and extravasation as compared to adults. In contrast, pre-activated tissue constructs were colonized by newborn monocytes at the same frequency than adult monocytes, suggesting that neonatal monocytes are capable of colonizing inflamed tissues. The presence of autologous plasma neither improved newborn homeostatic extravasation nor shaped age-specific differences in endothelial cytokines that could account for this impairment. Newborn monocytes demonstrated significantly lower surface expression of CD31 and CD11b, and mechanistic experiments using blocking antibodies confirmed a functional role for CD31 and CD54 in neonatal homeostatic extravasation. Our data suggests that newborn monocytes are intrinsically impaired in extravasation through quiescent endothelia, a phenomenon that could contribute to the divergent immune responsiveness to vaccines and susceptibility to infection observed during early life.

## Introduction

Divergent innate immune responses such as impaired extravasation of neutrophils to sites of local inflammation, can contribute to newborns known higher susceptibility to soft tissue and systemic infections^1–5^. Typically, divergent immunophenotypes are more extreme in the preterm than in the term newborn, associating with a particularly high risk for bacterial and fungal infections^6^. Neutrophils circulate in healthy individuals largely in a passive state with a very low efficiency to extravasate quiescent endothelia^7^. In contrast, circulating monocytes can escape the blood stream in the absence of inflammation under the low shear forces of capillary venules^8^, the smallest and most abundant veins consisting of a single-cell endothelial layer and a basement membrane or interstitium^9^. This constitutive extravasation enables a differentiation program that transforms monocytes into resident macrophages and dendritic cells (DCs) based on microenvironmental cues still not fully understood^10–14^; a biological process that asserts a key role to circulating monocytes in support of tissue homeostasis, infection clearance, vasculogenesis and overall immune-defence readiness^15,16^. Early in vitro studies suggested that in comparison to adults, human neonatal monocytes and macrophages exhibit no impairments related to adherence, random migration, chemotaxis, bactericidal activity, phagocytosis or production of oxygen intermediates (superoxide anion and hydrogen-peroxide)^17,18^. However, despite having normal random motility and adhesion capacity to extracellular matrix components in vitro^19–21^, neonatal monocytes demonstrate lower chemotaxis^19^ and activation potential by adjuvants^22^. Of note, although circulating newborn monocyte counts are equivalent to those of adults^23,24^, healthy human neonates demonstrate lower monocyte density in tissues as compared to adults^25,26^. Non-human primate studies revealed that tissue colonization by monocytes increases rapidly to adult levels within 1–2 days of birth, and cell recoveries from neonatal bronchoalveolar lavages suggest that a similar rapid increase takes place in humans after birth^27–29^. Since human newborns and infants are more susceptible to infections than adults, we postulate that this neonatal delay in transendothelial monocyte relocation into the tissues may contribute to their known window of vulnerability^30^. To better characterize the autonomous extravasation capacity of human newborn monocytes, we followed a tissue engineering approach to recreate the microanatomy and physiology of a human capillary vein and compared the extravasation of newborn and adult monocytes under more natural circumstances. Consisting of a confluent monolayer of primary human endothelial cells grown atop an extracellular matrix cushion, our three-dimensional (3D) vascular-interstitial interphase model enables the homeostatic translocation of purified monocytes through quiescent endothelia in the absence of exogenous cytokines^31^. Using this microphysiological tissue construct (TC) both the CD16− and CD16+ newborn monocytes demonstrated lower adherence and homeostatic extravasation through quiescent endothelia as compared to adult monocytes. In contrast, newborn monocytes extravasated pre-activated endothelium at the same frequency than adult monocytes, suggesting that the ability of newborn monocytes to colonize inflamed tissues is intact. Autologous plasma neither improved newborn homeostatic extravasation nor shaped age-specific differences in endothelial cytokines or chemokines that could account for this impairment. Flow cytometry analysis indicated significantly lower surface CD31 and CD11b on newborn monocytes, and mechanistic experiments using blocking antibodies confirmed a functional role for CD31 and CD54 in newborn homeostatic extravasation. Our findings indicate that neonatal monocytes are intrinsically impaired to extravasate quiescent endothelia but not inflamed ones, a distinct phenomenon that could contribute to the divergent immune responsiveness to vaccines and susceptibility to infection observed during early life.

## Results

### Newborn monocytes demonstrate lower adherence and homeostatic extravasation as compared to adult monocytes

**Figure 1:**
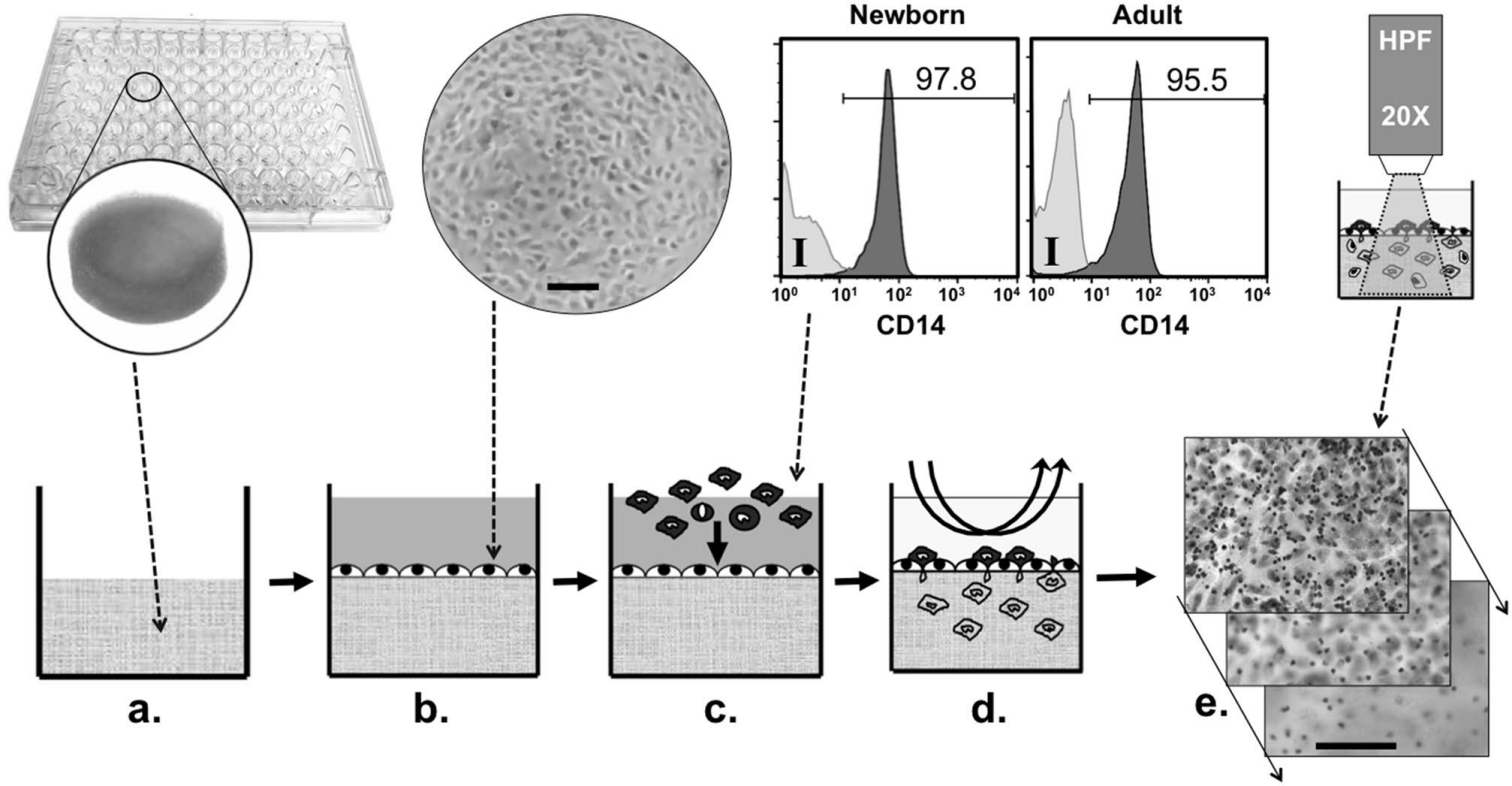
3D vascular-interstitial model. (**a**) Type I collagen is cast into cushions in a 96-well microtiter plate; (**b**) Single-donor human primary endothelial cells are grown over the cushions until achieving confluency (representative image); (**c**) CD33-purified human monocytes (representative histograms for CD14 expression) are then added to confluent tissue constructs (TCs) to enable autonomous extravasation for 1.5 h at 37 °C/5%CO_2_; (**d**) non-migrated cells are then removed by gentle aspirations; (**e**) TCs are then examined by microscopy or digested to extract extravasated monocytes for further analysis. *I* Isotype control. *HPF* High-Power Field. Scale bars = ∼200 μm.

**Figure 2:**
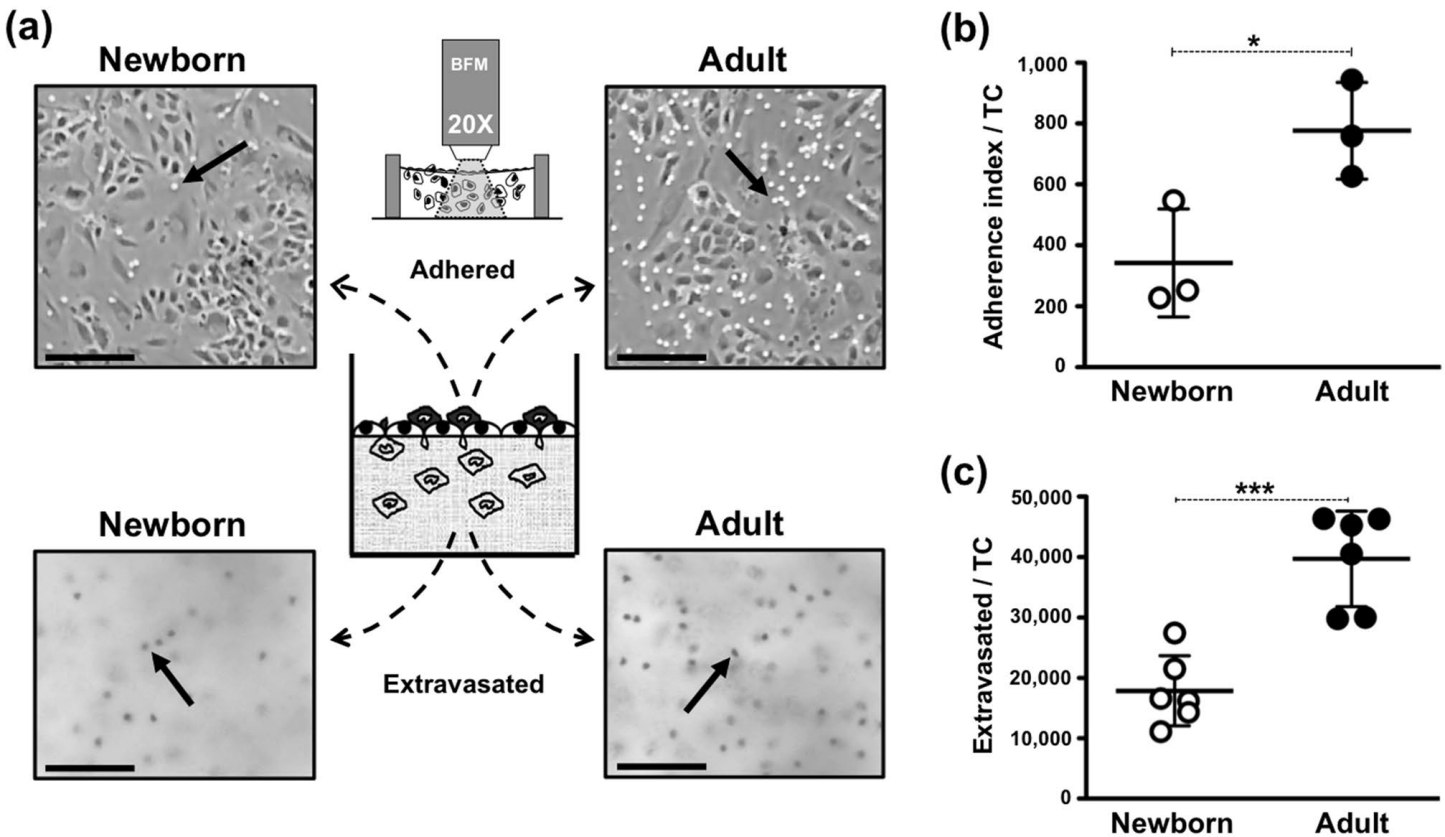
Newborn monocytes demonstrate lower adherence and extravasation as compared to adult monocytes. Purified human monocytes from either a newborn or an adult study participant were applied to each confluent TC well to enable autonomous extravasation under corresponding 100% autologous untreated plasma for 1.5 h at 37 °C/5%CO_2_, as described in methods. (**a**) Newborn and adult monocytes adhered to the endothelium (bright dots indicated by arrows) and extravasated (dark dots indicated by arrows) after removal of non-migrated cells from the top of TCs (representative images taken at surface level and at ~ 80 mm inside the constructs). Scale bar = ∼200 μm. (**b**) Index of adherence to quiescent endothelium, obtained as described in “Methods” (N = 3/age-group). (**c**) Number of viable extravasated monocytes from digested TCs (N = 6/age-group). Unpaired t-Test, Two-tails * = P ≤ 0.05; *** = P ≤ 0.005.

To our knowledge, the capacity of human newborn monocytes to extravasate has not been previously examined. Considering that venous endothelia regulates the relative abundance of tissue-resident monocytes in vivo during both inflamed and homeostatic colonization^9^, we recreated a quiescent vascular interstitium representing the components and architecture of capillary veins in vivo to assess neonatal monocyte autonomous extravasation under physiologically relevant conditions (i.e. autologous plasma). To this end purified monocytes, isolated through their myeloid marker CD33 from neonatal and adult mononuclear cells (MCs), were allowed to extravasate into our confluent quiescent tissue constructs at 37 °C/5%CO_2_ for 1.5 h under the presence of autologous untreated platelet-poor plasma (PP-plasma), in absence of exogenous cytokines. Non-migrated cells were removed and then constitutive adherence and extravasation of monocytes was measured as described in methods (Fig. 1). Results indicated that newborn monocytes adhered less abundantly to quiescent endothelial cells (Fig. 2a,b) as compared to adult cells (*P ≤ 0.05). Accordingly, the number of monocytes extracted from inside neonatal TCs was significantly lower (***P ≤ 0.005) as compared to adult TCs (Fig. 2c).

### Newborn monocytes are intrinsically impaired to extravasate quiescent but not inflamed endothelia

**Figure 3:**
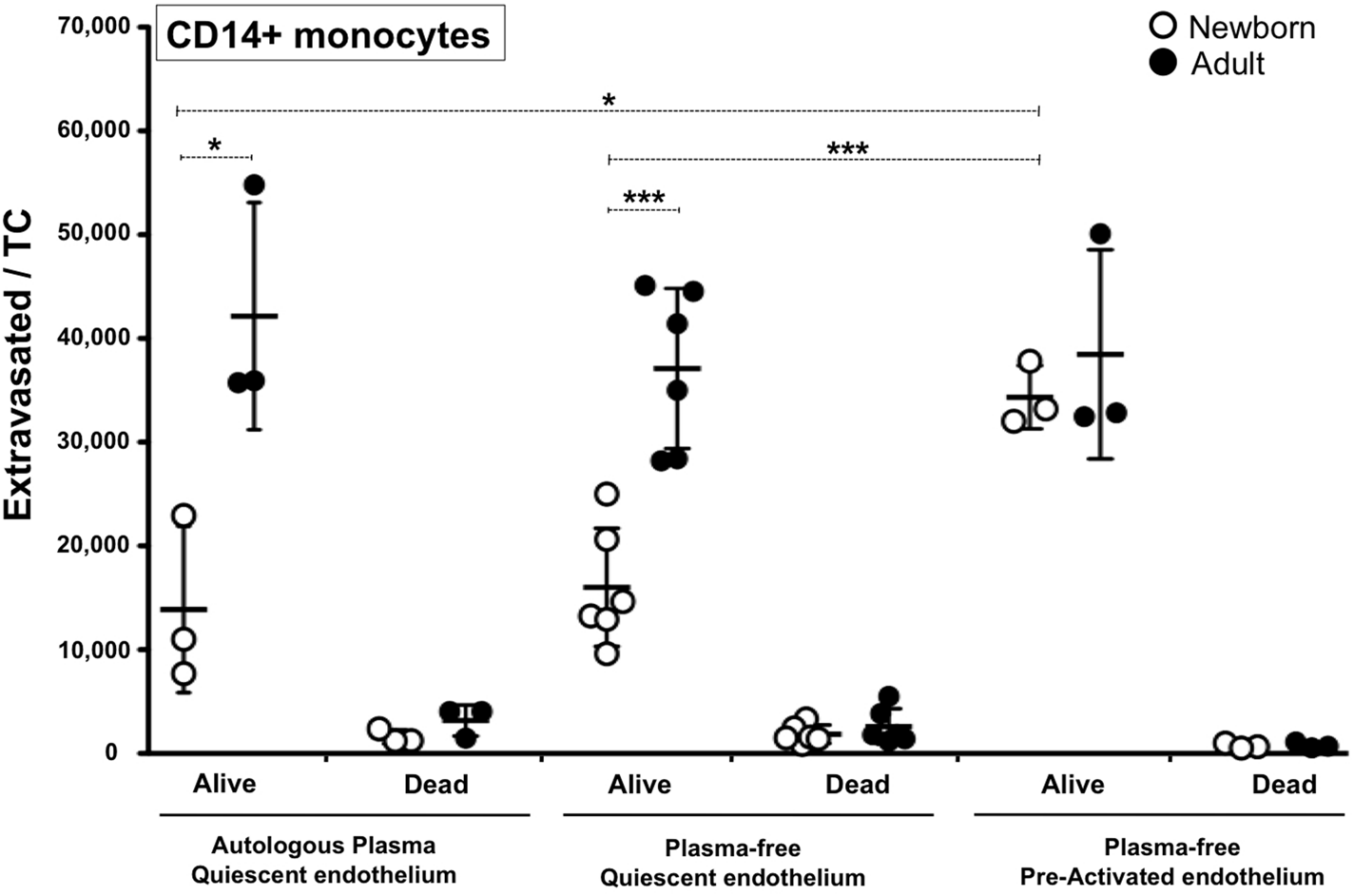
Newborn CD14 + monocytes are intrinsically impaired to extravasate quiescent but not inflamed endothelia. Monocytes were extracted from TCs after 1.5 h extravasation at 37ºC/5%CO_2_ under either autologous plasma on quiescent endothelium, plasma-free on quiescent endothelium or plasma-free on pre-activated endothelium conditions, as described in methods. Pre-activation of endothelium was done with a cocktail of TNF-α, IL-1β, IL-6 and PGE_2_ for 1 h (37 °C/5%CO_2_). Extravasated monocytes were analysed by multicolour flow cytometry and the number of extravasated CD14 + monocytes (Cells/TC) was calculated by combining viable cell counts with the percentages of viable monocytes detected by flow cytometry (N = 3–6/age-group). Two-tailed Paired t-Test compares same study participant between conditions. Two-tailed UnPaired t-Test compares age groups. n.s. = non-significant; *P ≤ 0.05; ***P ≤ 0.005.

**Figure 4:**
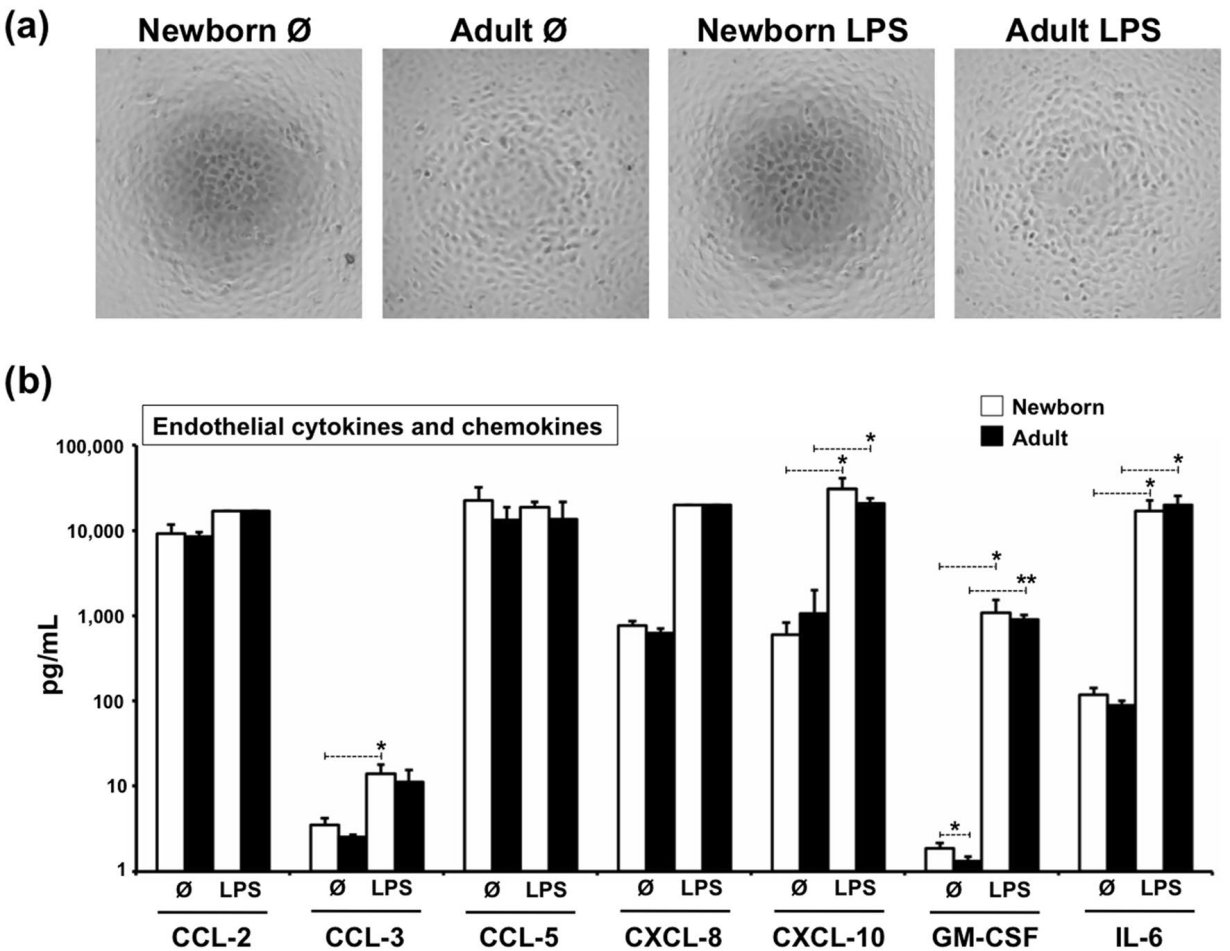
Cytokines and chemokines released by endothelium in the presence of autologous plasmas. Cytokines and chemokines were measured on study participant’s autologous plasmas (N = 3/age-group) after being over either quiescent or stimulated TCs devoid of monocytes for 12 h at 37 °C/5%CO_2_. 1 mg/mL LPS was used to stimulate TCs for 12 h, as described in methods. Unstimulated controls (Ø) received 1% DMSO. (**a**) Microscopic analysis of endothelium after stimulation (representative images, 4X). (**b**) Analytes measured were IL-1β, IL-6, CXCL8 (IL-8), IL-10, IL-12p40, IL-12p70, TNF-α, IFN-α2, IFN-γ, CXCL-10 (IP-10), CCL-2 (MCP-1), GM-CSF, CCL-3 (MIP-1α) and CCL-5 (RANTES). Chemokines CCL-2 and CXCL-8 in stimulated group reached their maximum detection limit. Cytokines IL-1β, IL-10, IL-12p40, IL-12p70, TNF-α, IFN-α2 and IFN-γ were not detected in both age groups. UnPaired t-Test, two-tailed *P ≤ 0.05; **P ≤ 0.01.

As neonatal plasma is particularly rich in age-specific immunomodulatory factors^30,32^ we evaluated homeostatic and inflamed monocyte extravasation under plasma-free conditions (Fig. 3). Our studies demonstrated that the presence of any soluble immunomodulators in cord blood plasma, including any potential residual anesthetic administered for C-section, was inconsequential to the observed impaired extravasation of newborn monocytes. Activation of the endothelium, a hallmark of inflamed tissues in vivo^33^, was performed for 1 h before letting monocytes extravasate for 1.5 h, as described in “Methods”. Extravasated monocytes extracted from digested TCs under these conditions were counted and analysed by multicolour flow cytometry. Viable extravasated cell counts per TC were combined with flow cytometry data using CD14 as a reliable lineage marker for circulating monocytes^34^. Resulting numbers of viable extravasated CD14 + cells per TC suggest that newborn monocytes are intrinsically impaired to extravasate quiescent but not inflamed endothelia. Cytokines and chemokines secreted by quiescent and stimulated single-donor endothelium in the presence of each donor’s autologous PP-plasmas (Fig. 4) support the notion that plasma is not contributing to the extravasation impairment. With the exception of GM-CSF being slightly higher in newborns, no significant age-differences were observed for any of the other 13 cytokines and chemokines measured. LPS dose used to activate TCs was sufficient to induce higher levels of GM-CSF, CXCL-10, CCL-2, CCL-3 and pro-inflammatory cytokines IL-6 and IL-8 for both age groups, without disturbing endothelial integrity (Fig. 4a). Cytokines IL-1β, IL-10, IL-12p40, IL-12p70, TNF-α, IFN-α2, IFN-γ were not detected and CCL-5 showed high levels independently of LPS stimulation (Fig. 4b). Overall, our results suggest that homeostatic impairment of neonatal monocytes to extravasate is intrinsic to the cells and not associated to humoral factors present in autologous PP-plasma or influenced by age-specific differences in cytokines or chemokines released by unstimulated endothelia.

### Both CD16+ and CD16− newborn monocytes demonstrate same intrinsic homeostatic impairment to extravasate and display lower surface expression levels of functional anchoring molecules

**Figure 5:**
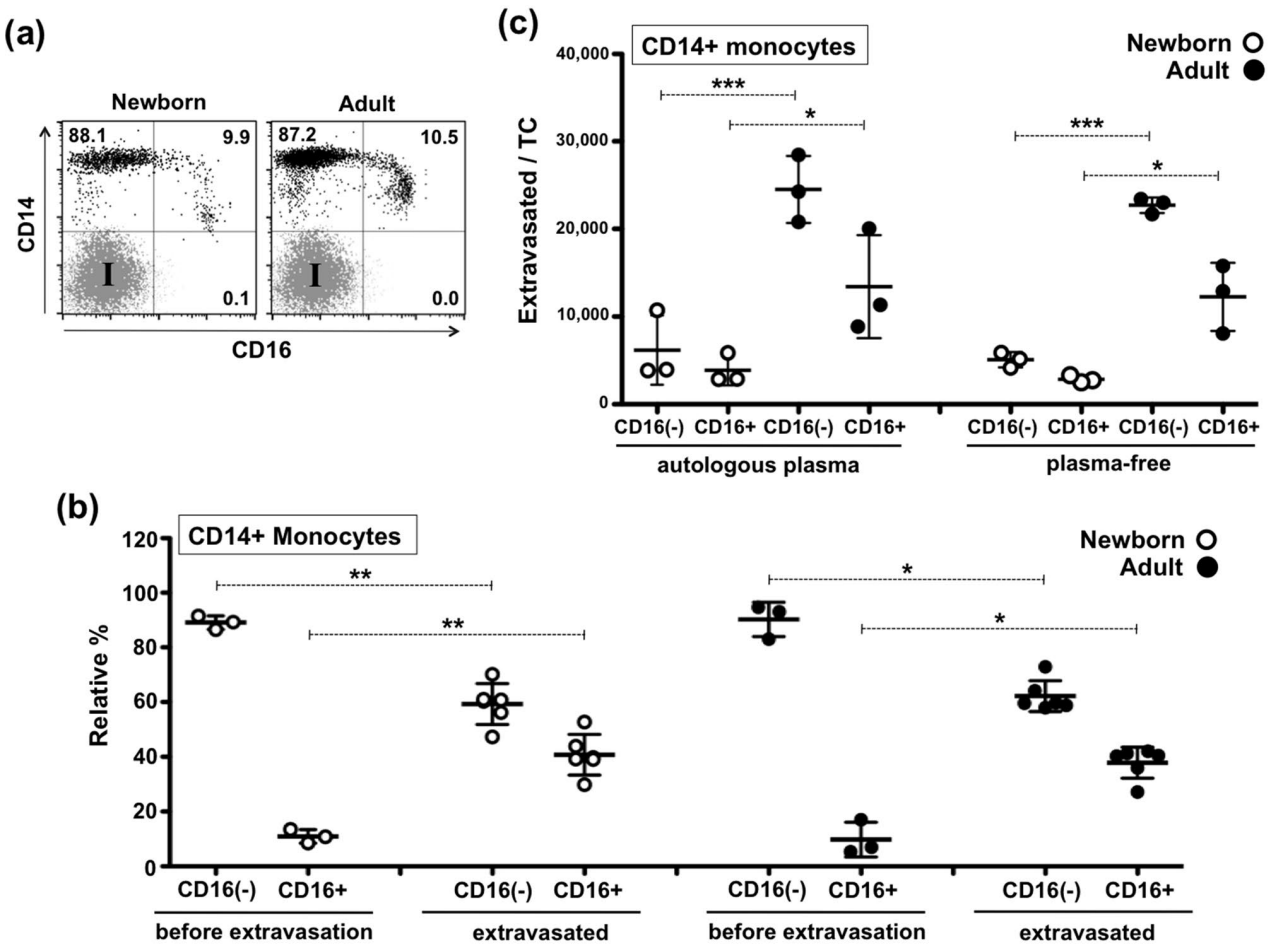
CD16+ and CD16− newborn monocytes demonstrate similar intrinsic homeostatic extravasation impairments. (**a**) Equal relative frequency of circulating CD14+++/CD16− and CD14dim/CD16+ on newborn and adult CD33-selected monocytes (representative dot plots). (**b**) Relative frequency of CD14+++/CD16− and CD14dim/CD16+ cells before and after plasma-free homeostatic extravasation demonstrate same relative enrichment of CD14dim/CD16+ monocytes inside newborn TCs and adult TCs. Viable extravasated monocytes per TC were counted before undergoing multicolour flow cytometry analysis for surface markers: HLA-DR, CD14, CD16, as described in methods. Percentages of CD16+ and CD16− monocytes equal 100%. (**c**) Both CD16+ and CD16− newborn monocytes demonstrate same intrinsic homeostatic impairment to extravasate as compared to adult counterparts. Autologous plasma has no effect on homeostatic extravasation of newborn and adult CD16− and CD16+ monocytes. The number of extravasated CD16− or CD16+ monocytes per TC was calculated by combining viable extravasated monocyte counts from digested TCs with percentages of viable CD14+++/CD16− or CD14dim/CD16+ monocytes detected by flow cytometry. I = Isotype control. N = 3–7 donors per age group. Paired t-Test, two-tailed was used for comparisons between same donors. UnPaired t-Test, two-tailed was used to compare age groups. *P ≤ 0.05; **P ≤ 0.01; *** = P ≤ 0.005.

**Figure 6:**
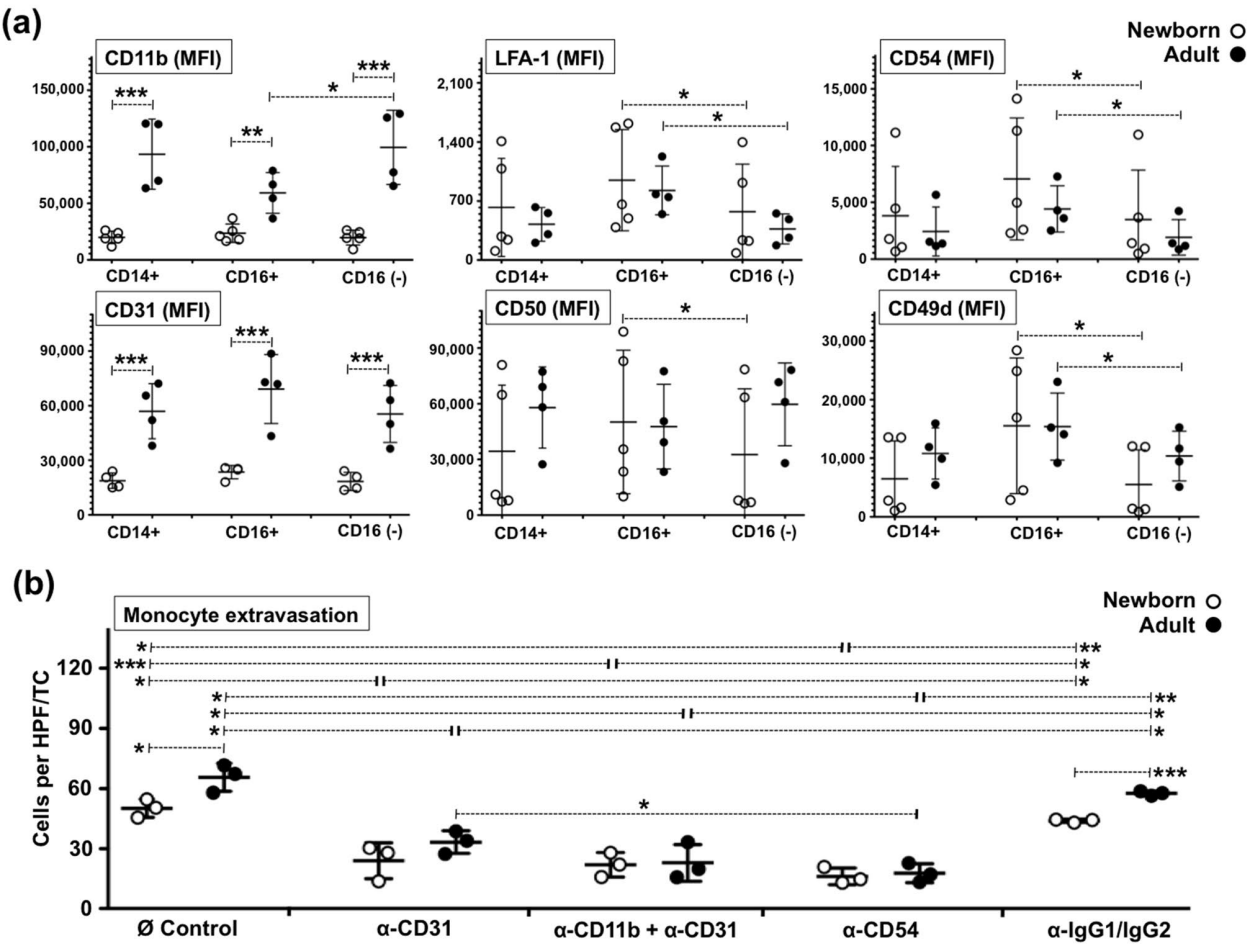
Neonatal monocytes express lower levels of functional CD11b and CD31. (**a**) The surface expression (MFI) of CD11b, CD31, CD54, CD50, CD49d and LFA-1 was studied on fresh newborns and adult monocytes using flow cytometry and analysed as total, CD16− and CD16+ monocytes (N = 4–5/age-group). *MFI* Mean Fluorescent intensity. (**b**) endothelium, as described in methods. *HPF* High-Power Field. Paired t-Test, two-tailed was used for comparisons between same donors. UnPaired t-Test, two-tailed was used to compare age groups. *P ≤ 0.05; ***P ≤ 0.005.

CD16 (FcgammaRIII) defines the largest known subpopulation of circulating human monocytes and since adult CD16+ monocytes are known to be preferential extravasators and DC precursors in a vascular model^35^, we assessed whether the extravasation impairment of neonatal monocytes was associated with this subpopulation of CD16+ monocytes. As previously reported^36^, our flow cytometry analysis indicated that the relative frequencies of peripheral CD14+++/CD16− (~ 88%) and CD14-dim/CD16+ (~ 10%) newborn monocytes are similar to those of adults (Fig. 5a,b). Consistent with previous observations for adult monocytes ^35^, homeostatic extravasation of CD14-dim/CD16+ monocytes was also relatively enriched inside TCs (Fig. 5b) as compared to CD16− monocytes. Nevertheless, when the actual number of extravasated CD16+ and CD16− monocytes was enumerated and analysed in comparison to their adult counterparts, both CD16+ and CD16− newborn monocytes demonstrated the same intrinsic impairment to extravasate (Fig. 5c). Of note, the homeostatic impairment of both monocyte subtypes appeared again to be independent of the presence of autologous newborn plasma. To further characterize the extravasation potential of human neonatal monocytes, we measured the cell surface expression of CD11b, CD31, CD49d, CD54, CD50 and LFA-1. These molecules represent the most ubiquitously reported adhesion molecules used by human monocytes to extravasate^37–43^. Among these markers, CD31 and CD11b demonstrated significantly lower levels on both the CD16− and CD16+ neonatal monocytes, as compared to their adult counterparts (Fig. 6a). Adult CD16− monocytes showed higher CD11b and lower LFA-1 and CD54, as compared to their CD16+ adult counterparts. Considering the lower expression of these anchoring molecules, we performed mechanistic experiments using blocking monoclonal antibodies (mAbs) to evaluate their functional participation in the homeostatic quiescent extravasation of newborn and adult monocytes. Blocking newborn CD31 marker significantly inhibited monocyte extravasation as compared to both the untreated and the IgG1/IgG2 isotype controls. The addition of anti-CD11b had no effect on the lower neonatal extravasation observed with anti-CD31, whereas for adult monocytes only the anti-CD31/anti-CD11b combo enabled significantly lower levels of extravasation. Significant inhibition of extravasation was achieved by anti-CD54 on both neonatal and adult monocytes (Fig. 6b).

## Discussion

Monocytes are essential for host defence through their capacity to detect, phagocytise and kill pathogens, and coordinating the generation and resolution of inflammatory immune responses and the maintenance of tissue homeostasis^6,8,15,16^. Despite similar monocyte counts in circulation^23,24^, healthy human neonates demonstrate lower monocyte density in tissues as compared to adults^25,26^ and, in contrast to neutrophils^1–5^, little is known about the ability of human neonatal monocytes to colonize healthy tissues. Herein, we defined for the first time the autonomous ability of human neonatal monocytes to extravasate across quiescent and inflamed endothelia under the presence of autologous untreated plasma using a human vascular interstitia model. The lower adherence and extravasation demonstrated by neonatal monocytes under this homeostatic microphysiological condition suggested the existence of an ontogenic impairment similar to that reported for neonatal neutrophils^1–5^. To assess whether newborn plasma, that is distinct from that of adults with respect to relative concentrations of soluble immunomodulators^30^ and the potential presence of residual epidural anesthesia, was contributing to the observed phenotype we repeated the experiments under plasma-free conditions. These studies indicated that the homeostatic impairment in neonatal monocyte extravasation was independent of autologous plasma. Pre-activation of endothelia with pro-inflammatory cytokines markedly increased neonatal monocyte extravasation to a level comparable to that of adults. Our results are consistent with in vivo observations that suggest that despite a lower homeostatic colonization of healthy tissues^25,26^, newborn monocytes demonstrate a rapid and efficient capacity to infiltrate tissues in response to inflammation^44^.

Using a similar human vascular model to the one employed in our current study, we have previously reported that adult monocytes expressing CD16 (CD14dim/CD16+) exhibited a more efficient capacity to extravasate quiescent endothelia than those lacking CD16 (CD14+++/CD16−)^35^. Of note, these same adult monocyte subsets have shown distinct immune capabilities^34^, DC differentiation^45^ and extravasation potential^33^. In this context, we considered whether a difference in the number of CD16+ monocytes and/or in their extravasation capacity could account for the observed impairment in newborn monocyte extravasation. As previously reported for healthy neonates^36,46–49^ and adults^45^, our phenotypic analysis of CD33-selected monocytes from our study participants (Fig. 5a) indicated similar relative frequencies of CD16+ and CD16− monocytes between newborns and the adults. Recently, Damasceno et al. studied the blood distribution of human monocyte subsets throughout life, classifying them as CD14+++/CD16−, CD14 + /CD16+ and CD14dim/CD16+^46,50,51^. In agreement with our observations, despite observing ontogenic trends in the distribution proportion of these three monocyte subsets throughout age, they reported no statistical significant differences between healthy cord blood, peripheral neonatal blood and adult blood (< 30 years of age)^46^. As reviewed by de Jong et al.^6^, there are contradictory studies with regard to the relative frequencies of adult and neonatal CD16+ monocytes^52–55^. It is possible that methodological differences (e.g. gating strategy, anticoagulant, monocyte selection, staining techniques) or even unanticipated human pre-existing conditions^56,57^ could account for some of these differences. The relative frequency of CD16− and CD16+ monocytes before and after plasma-free homeostatic extravasation confirmed that both adult and newborn CD16+ monocytes are preferential extravasators in our vascular model. However, despite applying the same number of monocytes to each well, when comparing the actual number of extravasated cells between age groups, both newborn monocyte subtypes demonstrated the same intrinsic impairment in homeostatic extravasation compared to their adult counterparts. Moreover, with the exception of GM-CSF, LPS-induced concentrations of cytokines and chemokines involved in activation, infiltration and differentiation of leukocytes, released by the endothelium in the presence of autologous plasma (but in the absence of monocytes), revealed no significant ontogenic differences. Of note, the increased levels of Monocyte Chemoattractant Protein-1 (MCP-1/CCL-2) released by stimulated endothelia, a chemokine acting on monocytes via CCR2, could contribute to the increased neonatal extravasation noted on stimulated TCs. However, newborn monocytes may express reduced levels of CCR2 relative to their adult counterparts^58,59^.

Our data suggests that newborn monocytes extravasate as efficiently as adult cells through inflamed endothelia but are intrinsically impaired to extravasate quiescent vasculatures. Considering that newborn monocytes could express a divergent set of surface anchoring molecules, used selectively to colonize healthy non-inflamed tissues, we assessed CD11b, CD31 (PECAM-1), CD49d, CD50 (ICAM-3), CD54 (ICAM-1) and LFA-1 (CD11a/CD18). These markers are important mediators of human monocyte extravasation^37–43^ and are constitutively expressed on the surface of monocytes^60^. CD49d pairs with β1-integrin CD29 to form VLA-4, and CD11a and CD11b couple with β2 integrin CD18 to form LFA-1 and CR3, respectively. These integrins bind to CD31, CD50 and CD54, present on the luminal side of the endothelial surface. Our flow cytometry analysis revealed a significant lower newborn surface expression of CD31 and CD11b on both the CD14+++/CD16− and the CD14dim/CD16+ monocytes, as compared to same adult subtypes. Adult CD14+++/CD16− monocytes demonstrated higher CD11b and lower LFA-1 and CD54, as compared to their own CD14dim/CD16+ counterparts. These results are in agreement with reports of reduced CD11b expression on cord blood monocytes^37,41,61^, including correlation with gestational age^6^, as well as those indicating increased CD31, CD54 and decreased CD11b levels on adult CD16+ monocytes as compared to their CD16− counterparts^62,63^. Of note, an increase of CD11b on circulating monocytes is a biomarker for late-onset sepsis in extremely low-birth-weight neonates^64^. This aspect seems in line with the higher extravasation observed by neonatal monocytes in our activated endothelia. Another report indicated a trend-down lower expression of CD31 on neonatal monocytes as compared to 1-month old infants and adults^51^. Methodological aspects such as monocyte selection, purity and heterogeneity or the use direct conjugated antibodies and cold temperature, could account for how our results managed to achieve statistical significance for CD31.

Lower expression of CD31 and CD11b suggested a functional correlation with observed impaired ability of newborn monocytes to extravasate quiescent endothelia. Of note, monocyte-endothelium homophilic CD31/CD31 interactions are not only important adhesive steps but they also trigger signalling events leading to increased CD11b on monocytes^65^. As with the case of neonatal neutrophils, cell surface levels of these molecules are not enough to presume functionality. While several studies had reported an increase^66^, equal^67–69^ or decrease^70–72^ in CD11b expression on infant neutrophils as compared to adults, the functional role of neonatal CD11b on impaired extravasation of neutrophils into inflamed tissues was demonstrated by using blocking mAbs^4^. Thus, using mAbs to block specific anchoring molecules we evaluated whether neonatal CD31 alone or in combination with CD11b, were functionally relevant for monocyte extravasation through quiescent endothelia. Anti-CD54 mAb was included as a positive control for adult extravasation, since adult CD14dim/CD16+ monocytes are preferential extravasators in this model^35^, exhibit higher levels of CD54, and the blockage of this molecule is known to impair adult monocyte extravasation^73^. Our results indicated a primary usage of CD31 by neonatal monocytes during homeostatic extravasation. For adult monocytes, while anti-CD31 mAb alone reduced quiescent extravasation, concurrent blockage of CD31 and CD11b demonstrated greater inhibition of extravasation. As expected, an anti-CD54 mAb diminished the extravasation of adult monocytes, serving as a positive performance control for our model. Neonatal monocyte extravasation was also inhibited by anti-CD54. These results indicate a functional role for CD31 and CD54 in the homeostatic extravasation of neonatal monocytes, and while the expression of CD54 was not significantly lower on the surface of newborn monocytes, this molecule is expressed also on endothelial cells and is an important anchor molecule for CD11b/CD18^73^. Of note, CD31 is a multi-functional adhesion and signalling molecule that displays both pro- and anti-inflammatory effects^65^; thus, deficient CD31 expression on neonatal monocytes may have wider implications for the regulation of early life immunity.

**Figure 7:**
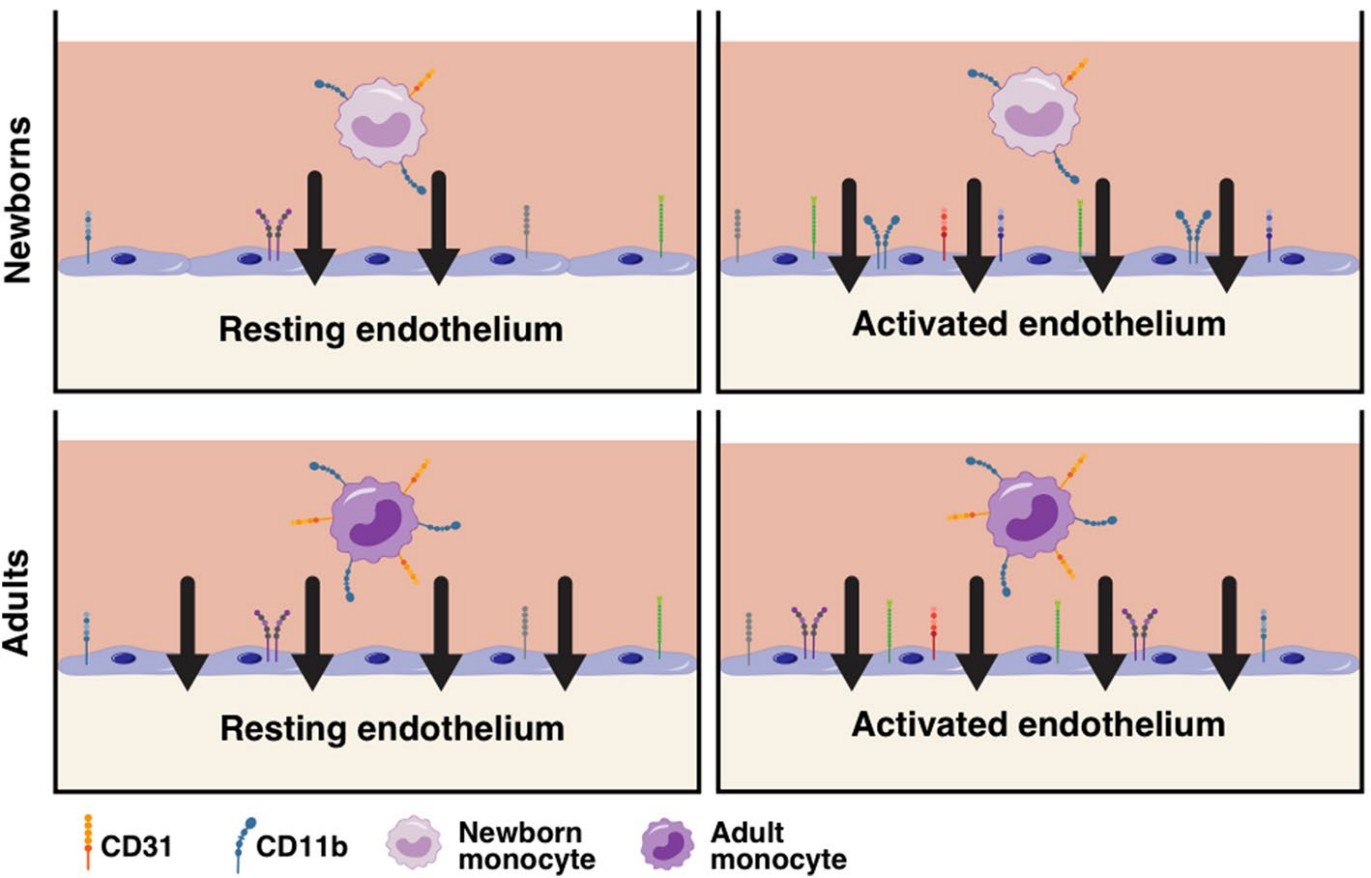
Neonatal monocytes demonstrate impaired homeostatic extravasation into a microphysiological human vascular model. To study the ability of monocytes to undergo autonomous transendothelial extravasation through a confluent human endothelium under biologically accurate circumstances we engineered a three-dimensional human vascular-interstitial model. Our results suggest that neonatal monocytes are intrinsically impaired to migrate through resting endothelia but maintain sufficient capacity to extravasate activated endothelia as efficiently as their adult counterparts. Newborn monocytes demonstrated significantly lower surface expression of CD31 and CD11b, and mechanistic experiments using blocking antibodies confirmed a functional role for CD31 and CD54 in neonatal homeostatic extravasation. To the extent this microphysiological model reflects the behaviour of monocytes in vivo, this phenomenon could contribute to the divergent immune responsiveness to vaccines and the susceptibility to infections observed during early life.

To the extent our microphysiological model reflects the behaviour of monocytes in vivo, our study suggests that while neonatal monocytes are intrinsically impaired to colonize quiescent tissues, when encountering an inflammatory environment, they can colonize tissues as efficiently as their adult counterparts (Fig. 7). This distinct cell-mediated phenomenon may reflect the demands of the foetal environment focused in avoiding potentially harmful inflammation in utero. Relatively low monocyte density in quiescent human neonatal tissues such as lungs and dermis^25,26^, coupled with impaired extravasation of newborn neutrophils to sites of local inflammation^1–5^, may contribute to the susceptibility of newborns to infection with pyogenic and intracellular microbes. Neonatal endothelial activation may limit local neutrophil infiltration, favouring efficient monocyte extravasation. Monocytic influx across activated endothelia may enhance host defence at infected sites, but with respect to aseptic inflammatory pathologies, such as hypoxia-reperfusion, could contribute to tissue injury including brain damage^44^.

Our study has several limitations, including (a) lack of microphysiologic fluid flow which can impact cell migration and phenotype^74^; (b) a focus on full term newborns, whereas preterm newborns are particularly susceptible to infections and its sequelae including long-term inflammatory organ dysfunction^6^; and (c) a focus on cord-, as opposed to peripheral-, blood plasma and leukocytes. Indeed, neonatal cord blood leukocytes may be distinct from peripheral blood leukocytes possibly reflecting epigenetic changes due to post-natal exposures, including acquisition of the microbiome. Limitations in the volume of peripheral blood that can be obtained from healthy preterm or term neonates < 28 days of age^75^ precluded this approach. While our microphysiologic model suggests the existence of a homeostatic impairment in extravasation of human neonatal monocytes, further studies are required to confirm this finding in vivo and better dissect the molecular mechanisms underlying it.

Overall, our study lends fresh insight into the distinct phenotype of newborn monocytes, uncovering a potential mechanism that may contribute to early life susceptibility to infection and impaired responses to vaccines. Our microphysiologic tissue construct platform represents a powerful translational tool that can now be used to study agents that inhibit monocyte migration, which may be useful for discovery and development of treatments for inflammatory disorders. Conversely, agents that locally activate neonatal monocytes and endothelial cells to induce robust monocyte migration into immunization sites may serve as effective vaccine adjuvants to shield this vulnerable population^76^.

## Methods

### Reagents

Culture media M199 and RPMI-1640, Hank’s Balanced Salt Solution (1X HBSS), Dulbecco’s Phosphate Buffered Solution without calcium or magnesium (1X DPBS), 0.25% Trypsin–EDTA (100X), ultrapure EDTA (0.5 M), Paraformaldehyde (16% PFA) and Penicillin/Streptomycin/Glutamine (100X PSG) were purchased from Gibco (Thermo Fisher Scientific Inc.). Pyrogen-free heparin was purchased from Sagent Pharmaceuticals (Schaumburg, IL). Collagenase enzyme with low tryptic activity was from Roche Diagnostics (Indianapolis, IN). Sodium hydroxide (NaOH 10 M), Dimethyl sulphoxide (100% ACS DMSO, Hybri-Max), Trypan Blue stain (0.2%), Haematoxylin–Eosin (H&E), Toll-Like Receptor (TLR)-4 agonist Lipopolysaccharide (LPS, 500,000 EU/mg) and Glucose-6-Phosphate (G-6-P) were obtained from Sigma-Aldrich (St. Louis, MO). Ficoll-Paque PREMIUM was from GE Healthcare Life Sciences (Pittsburgh, PA). Tumor Necrosis Factor alpha (TNF-α, interleukin (IL)-1 beta (IL-1β), IL-6 and Prostaglandin E_2_ (PGE_2_) were purchased from R&DSystems (Minneapolis, MN). Blocking mAbs were procured from Invitrogen (Carlsbad, California) and used at specific concentrations after proper titration. Anti-CD11b was used at a 1:100 dilution, anti-CD54 at 1:50 dilution, anti-CD31 at 1:10 dilution and corresponding IgG1 kappa and IgG2a kappa Isotype controls both used at 1:100 dilution.

### Human materials

Adult blood (50–300 mL) from healthy study participants was collected via peripheral venipuncture after written informed consent in accordance with the Declaration of Helsinki and as approved by the Institutional Review Board (IRB) of Boston Children’s Hospital (BCH) (protocol number X07-05-0223). Newborn blood (50–110 mL) was collected via umbilical cord venipuncture), immediately after elective Caesarean-section delivery (epidural anaesthesia) of de-identified mothers with no record of fever, HIV or other acute or chronic infections, following protocols approved by the local IRBs of The Brigham and Women’s Hospital (Protocol #2000P000117/BWH) and the Beth Israel Deaconess Medical Center (Protocol #2011P-000118/BIDMC). According to the clinical data collected at the time of C-sections, all of our umbilical cord blood collections were from full term neonates with a mean gestational age (GA) of 38.94 ± 0.5 weeks (range 37.6 to 39.5 weeks GA and a 1.5:1 female/male ratio). Blood samples were drawn into 60 mL syringes containing pyrogen-free heparin (final concentration 20 units/mL) and processed within 2 h of collection to separate plasma and MCs. Briefly, blood was centrifuged at 500×*g* for 10 min at 24 °C to separate plasma and hemocytes. Plasma was centrifuged again at 3000×*g* for 30 min at 24 °C to generate PP-plasma and then frozen at − 80 °C until further analysis. Plasma used on studies was never heat-treated. Hemocytes were resuspended in 1 × DPBS with 2%v/v heparin to regain the original blood volume. MCs were isolated by Ficoll density gradient centrifugation according to manufacturer’s specifications. Both PP-plasma and matching MCs (100 million/mL) were cryopreserved if not used immediately. Plasma-free cryopreservation media for MCs consisted of 1 × DPBS containing 10% DMSO, 44 mg/mL clinical grade Human Serum Albumin (HSA, Octapharma, Lachen, Switzerland) and 2 mM EDTA, as described earlier^31^. Human type I collagen solution (VitroCol, 3 mg/mL) was purchased from Advanced Biomatrix (San Diego, CA). Human Fibronectin was purchased from Biomedical Technologies Inc. (Stoughton, MA). A single-donor lot of Human Umbilical Vein Endothelial Cells (HUVEC) was purchased from Lonza (Walkersville, MD USA) and used across all of our experiments.

### Endotoxin testing

Kinetic Turbidimetric (KTA) LAL test, using the KTA2 reagent, was used to confirm all human materials and reagents were endotoxin-free (≤ 0.2 Endotoxin Units/mL at working concentration) in accordance with manufacturer’s instructions (Charles River Laboratories International, Inc.; Wilmington, MA).

### 3D vascular-interstitial model

Building from prior tissue engineering efforts^31,77^, our three-dimensional model consists of a confluent quiescent monolayer of human endothelial cells grown over a basement membrane of extracellular matrix components. Briefly, endotoxin-free type I collagen cushions were cast in 96-well microtiter plates (Costar round bottom, Thermo Fisher Scientific Inc.), cushions were pre-coated with a 0.5 mg/mL solution of human fibronectin, second passage single-donor HUVEC were seeded on top and cultured at 5% CO_2_/37 °C in M199 media containing 50% FBS and 1% PSG until reaching confluency, as described earlier^31,35^. Collagen cushions were prepared by mixing 10 × M199 media, 0.1 N NaOH and a 3 mg/mL collagen solution, at a proportion 1:5:8, respectively. When cultured in vitro over an extracellular matrix, HUVECs naturally and autonomously form a tight cobblestone monolayer, as demonstrated in Figs. 1 and 4. Monolayer integrity, confluence and morphology of TC endothelial cells were periodically evaluated with an inverted light microscope (EVOS XL CORE, Thermo Fisher Scientific Inc.) prior to initiating assays. All of our experiments employed the same single donor HUVEC cells, grown at same time, with same media, materials and incubator, and TCs were used for testing only after reaching 100% endothelial cell confluency (Fig. 1). While our vascular tissue constructs are innovative in that they are entirely human and age-specific, the tissue engineering principles of culturing HUVEC on top of the extracellular matrix cushions are based on those we have previously reported^31^.

### Monocyte extravasation assay

All monocytes used in our study were positively selected from MCs using magnetic microbeads covalently linked to anti-CD33 mAbs (MACS, Miltenyi; Cambridge, MA), following manufacturer recommendations. Resulting purities assessed by CD14 expression normally achieved > 95% (Fig. 1). CD33 was chosen as a broad myeloid selection marker instead of CD14 in order to: (a) avoid interference with important antigen-capture molecules^78^; (b), avoid induction of non-physiological activation^79^; and (c), ensure the natural monocyte heterogeneity based on the expression levels of CD14 and CD16 (i.e. including the CD14low/CD16+)^45^. All of our experiments employed the same single donor HUVEC cells, grown concurrently, with standardized media, materials and incubator as previously described^31^. After verifying confluency, tissue constructs were used to test newborn *versus* adult monocyte extravasation side-by-side, comparing monocytes derived from one participant from each group (i.e., one newborn and one adult) per experiment. Endothelial confluency was verified in every well on each day of the experiment using an inverted microscope. 10^5^ CD33 + monocytes from either a newborn or an adult were applied to each confluent TC well to enable their autonomous extravasation under motionless conditions for 1.5 h at 37 °C/5%CO_2_. Monocyte extravasation was studied under 100% autologous heparinized untreated PP-plasma, a natural source of age-specific immunomodulatory factors^30,32^, and under plasma-free M199 media containing 1% PSG and 0.1% HSA. In some experiments the endothelium was pre-activated at 37 °C/5%CO_2_ with either Lipopolysaccharide (LPS at 1 μg/mL) for 12 h or with a human cytokine cocktail commonly used to mature autologous monocyte-derived DCs for clinical trials^80^ consisting of TNF-α (10 ng/mL), IL-1β (2 ng/mL), IL-6 (10 ng/mL) and PGE_2_ (1 μg/mL) for 1 h. These inflammatory mediators have been used before to pre-activate endothelial cells in leukocyte extravasation studies^81–83^. Pre-activated TCs had their endothelium washed several times with warm HBSS 1X to remove stimulants and were also inspected microscopically for signs of confluence disruption before testing monocyte extravasation. In all cases, each experiment included three replicates per condition and non-migrated monocytes were removed after the 1.5 h incubation period by gentle aspiration and several consecutive washes with warm 1 × DPBS (Fig. 1).

### Assessment of extravasated/adhered monocytes

Migrated and adherent monocytes (Fig. 2a–c) were retrieved from TCs by ECM digestion using collagenase, according to manufacturer’s recommendations. Liquefied TCs were passed through a 50 μm pore-size cell-strainer to remove large endothelial sheets, leaving freed monocytes to be counted using Trypan-Blue vital-staining (Fig. 2c). In some cases, monocyte extravasation was rapidly determined using microscopy on 4% PFA fixed TCs, as described earlier^84^. For this, extravasated monocytes in focus at 30 µm beneath the endothelium were counted in at least five centred-High-Power Fields (HPF) on several technical replicas using 20X objective (Figs. 1 and 6b). This practical extravasation index was previously validated with paralleled cell counts from digested autologous TC replicas. Monocyte adherence was obtained from microscopy pictures of TCs at endothelial focal plane (10X) for 3 neonates and 3 adults. Adhered monocytes (bright dots) were counted for same area at the centre of each TC well (Fig. 2b) after removal of non-migrated cells. Phenotypic analysis of selected CD33+ monocytes was performed using polychromatic flow cytometry. Briefly, cells were stained at 4 °C/dark for 30 min with direct-conjugated mAbs against CD14-APC, CD16−PE, HLA-DR-FITC, CD50-BV650, CD49d-BV650, CD54-BV650, CD11b-BV421, LFA1-BV421 and CD31-BV421 (BD Biosciences), as recommended by manufacturer. Stained cells were rinsed with DPBS and fixed with freshly made cold 4% PFA solution. Three overlapping panels per sample were ran consecutively on same equipment: Panel 1: HLA-DR-FITC, CD14-APC, CD16−PE, CD31-BV421 and CD50-BV605; Panel 2: HLA-DR-FITC, CD14-APC, CD16−PE, LFA1-BV421 and CD54-BV605; Panel 3: HLA-DR-FITC, CD14-APC, CD16−PE, CD11b-BV421 and CD49d-BV605. Test-mAbs were excess/titrated and corresponding direct-conjugated isotypes and unstained controls were used to define unspecific fluorescence. Minus-one-fluorochrome controls were used to compensate fluorescent overspill in a BD LSR-Fortessa instrument (Becton Dickinson). Equal numbers of events were collected for each sample. Data was analysed with FlowJo Software (Tree Star, Inc.; Ashland, OR) employing a commonly used single Forward- *versus* Side-Scatter (FSC *versus* SSC) viable-gate to remove debris events based on size and granularity^45^. The percentage of viable events for a desired marker was used to calculate the number of viable extravasated monocyte subtypes (i.e. CD16+ monocytes) per a single TC well; for this, the number of viable monocytes per TC, extracted by collagen digestion was multiplied by the percentage of viable positive events obtained by flow cytometry and then divided by 100.

### Cytokines/chemokines released by endothelium

TCs containing no monocytes but only PP-plasmas were stimulated with bacterial LPS (1 μg/mL) or left unstimulated (1% DMSO) for 12 h at 37 °C/5%CO_2_. After this, supernatants were collected and either cryopreserved or immediately analysed for the release of IL-1β, IL-6, CXCL8 (IL-8), IL-10, IL-12p40, IL-12p70, TNF-α, IFN-α2, IFN-γ, CXCL-10 (IP-10), CCL-2 (MCP-1), GM-CSF, CCL-3 (MIP-1α) and CCL-5 (RANTES) using a fluorometric bead-based array Multiplex kit (Millipore; Billerica, MA) and a Luminex Multiplex Instrument (Millipore), following manufacturer’s recommendations. Supernatants were cryopreserved at − 80 °C until analysed as both neat and 1:20 dilution after a single freeze–thaw cycle. For comparison purposes, cytokines reaching the maximum detection limit were arbitrarily assigned with the maximum detected on their respective standard curves. Since the use of a cytokine cocktail containing IL-1β, IL-6 and TNF-α would have interfered with these readouts, LPS was chosen to stimulate the endothelium, as previously reported^85^.

### Assessment of monocyte extravasation in the presence of blocking mAbs

Some experiments included conditions where monocytes were independently incubated with titrated endotoxin-free blocking mAbs against CD11b, CD54 and CD31 for 30 min (4 °C) before extravasation, as commonly reported^4,86^. Equal numbers of incubated monocytes, including their plasma-free media containing the corresponding blocking antibody, were applied over quiescent TCs to extravasate for 2 h. Corresponding IgG1 kappa and IgG2a kappa antibody isotypes were used as specificity controls. An unstimulated control (Ø) was also included receiving only a volume of sterile aqueous carrier solution equal to corresponding mAb tested or, when several doses were tested, the volume corresponding to the highest concentration tested for that mAb.

### Statistical analysis and graphs

Typically, each assay included one newborn and one adult, tested side-by-side for each condition with multiple technical replicas. Descriptive statistics are reported as the mean of results produced by each study participant ± standard deviation. Error bars represent standard deviations. Statistical analyses were performed using GraphPad Prism 4 software (GraphPad Software Inc.) or Excel:Mac (Microsoft Excel). The statistical test used was the Student’s t-test, two-tailed paired to compare matched observations and two-tailed unpaired for observations between study participants. Only P values ≤ 0.05 were considered statistically significant and typically denoted in figure legends as *P ≤ 0.05, **P ≤ 0.01, and ***P ≤ 0.001. Images and graphs were generated using FlowJo Software (Tree Star, Inc.; Ashland, OR), Excel:mac (Microsoft Excel) and PowerPoint:mac (Microsoft Excel).

## Data Availability

The datasets supporting the conclusions of this study will be made available by the authors, without undue reservation, to any qualified researcher. Per HIPC data guidelines, some data has been deposited to NIAID’s *ImmPort* website (https://immport.niaid.nih.gov/home) under study accession number SDY1659.
